# Transactions of the Medical and Physical Society of Calcutta

**Published:** 1834-04-01

**Authors:** 


Transactions of the Medical and Physical Society of Calcutta. Vol. VI., pp. 509. Calcutta, 1833. Parbury and Allen, London, November, 1833.

The arts, as well as the sciences, are progressing on the banks of the Ganges. Those who remember the specimens of typography which the Calcutta and Madras press sent forth 30 years ago, will be astonished at beholding the sixth volume of the Transactions, now before us—a volume that would be no discredit to some of the best steamers that facilitate the march of intellect in this great Metropolis! The medical sciences, too, are evidently keeping pace with the arts throughout our vast Asiatic Empire, as the contributions to these volumes, from various provinces of that wide-spread country, abundantly prove. The members of the Medical and Physical Society of Calcutta are now, we imagine, more numerous than those of any medical society of Europe—or at least of the mother country. The present volume contains twenty-five papers, and an appendix in small type, presenting 30 additional articles—so that there is here a very great variety of information—much of interesting import even to the members of the profession in these western regions. Our intercourse with the East, which will now be greater than ever, demands from us a notice of these volumes as they issue from the press, and we shall accordingly proceed to our task.

I. ON THE CLIMATE OF BANGALORE, AND THE PREVALENCE OF HEPATITIS AT THAT STATION. By *J. Mouat*, M.D.

Previously to the establishment of a salutarium in the Nilgherries, Bangalore was considered the Montpellier of Madras—and it still maintains its character, though a drawback on its good name appears in the sequel of this paper. In January, the thermometer ranges from 62° to 71°—in April, from 75° to 87°—in July, from 69° to 79°, weather cool and sun generally obscured—in December, " the air very cold, elastic, and bracing—the sky cloudless—wind strong from the North and North-east—thermometer from 68° to 72°, very few sick."

Bangalore is a table-land, nearly 3000 feet above the level of the sea, enclosed by the Eastern and Western Ghauts, and its climate is comparatively cool. The aspect is generally barren and jungly.

" The air is always elastic, and generally pleasant, even in the hot season; and during the monsoons moist, but not relaxing or disagreeable, though at times cool and chilly. Too much can scarcely be said in praise of the climate of Bangalore: it is truly excellent, and convalescence most rapid even from acute disease; neither does it seem to predispose to phthisis,* hooping-cough, or croup, nor to retard the recovery of those affected with syphilis, or occasion intermittents. In the jungles, and at Seringapatam, remittent fevers are prevalent, but these arise from local causes. The coolness may not prove favourable to asthmatic subjects, or delicate persons, who are easily affected by changes of temperature and so rarefied an atmosphere; or those who are labouring under organic deranged structure, and certain idiosyncrasies of constitution; but to all others, it must be a delightful and happy change from the heat of the Carnatic, or the relaxing effects of Malabar, and of most other Indian stations." 11.

It was not to be expected that, in such a climate, hepatitis would have been very prevalent—yet such is the case, and it is attributed by the author of the paper to exposure to the sun and intemperance. Dysenteries, fevers, hepatic affections, and rheumatisms, were the prevailing acute diseases in the cool months. The following was the treatment employed in dysentery.

" DYSENTERY. This has been a highly acute affection, and treated as such, by copious and repeated venesections, leeches to the abdomen and the perinæum, large or scruple doses of calomel, often combined with ipecacuanha, and the latter sometimes by itself, or with gentian, or the blue pill. Frequent mild purgatives, especially the Ol. Ricini. Hot fomentations and the hip bath, emollient or ipecac. injections, blisters, &c." 23.

FEVERS. Few died of fevers at Bangalore, although they were fully as violent and frequent as dysentery. Out of 121 cases of fever, only two died. The treatment was strictly antiphlogistic.

HEPATITIS. Medical writers, among others Drs. Annesley and J. Johnson, have considered hepatitis as, in some respects, endemial in certain localities, owing probably to some peculiarity of the soil and the seasons. It is difficult, however, to account for the fact, that hepatitis is so frequent in Bangalore—a climate so much superior to many others in the peninsula of

* " In nearly 13 years, only two men have died of consumption in the regiment."

India, where the disease is much less frequent. The annual mean temperature is 74° Fahr., a very low range for India. It is curious that, in Bangalore, hepatitis never affects children, nor natives, and very seldom females. This last circumstance our author thinks must upset the theory of high temperature being the cause of hepatitis. But it is to be recollected that women are less exposed than men to the general causes of diseases, and that it is not high temperature alone that causes hepatitis. Great heat predisposes the constitution to be acted on by cold, and it is to transitions of temperature, rather than to a very high or a very low range of it, that man owes most of his maladies, in India and in other climates.

### II. A Sketch of the Medical Topography of Gowhattee, with an Account of the prevailing Diseases, &c. By *John Leslie*, Esq.

This is the capital of Assam, on the southern bank of the Burrampooter, and in the 26th parallel of N. Latitude, and about 500 miles from Calcutta. In consequence of the annual rise of the great river, two thirds of the ground is inundated in the wet season. The range of the thermometer is from 80 to 86, and the nights are generally cool.

"The peculiar manufacture of the opium deserves a few remarks. The white variety of poppy is most generally cultivated; it ripens in February, and the exudation from the wounded capsule is wiped off with a piece of old rag. This when saturated is rolled up and successive layers applied, until a mass be obtained of about a seer in weight. The opium in this state is called *kanee,* and to its use, or rather abuse, the Assamese are unfortunately exceedingly addicted. The drug is used in two modes, one method is by daily dipping a portion of the rag in water and drinking the solution; the other is, to strain and evaporate the solution, so as to obtain a pure extract of opium, which those who can afford it smoke. The latter mode is chiefly confined to the higher classes, and is similar to the practice of the Chinese." 39.

The population of Gowhattee is about sixty thousand. The diet of the poor is very meagre, consisting of rice, seasoned with a kind of alkaline salt or potash. Our author's experience was almost exclusively confined to the jail, a large building well situated on the banks of the river. The ordinary diseases are fevers, bowel-complaints, dropsy, ulcers, syphilis, and cutaneous affections. The following passage is deserving of notice.

"Fevers often usher in that intractable form of bowel complaint, from which so large a proportion of the mortality of this jail originates. This disease has its seat in the great intestines, and its approach is frequently overlooked; and emaciation to a considerable extent occurs before the patient applies for admission into hospital. In not a few cases, a patient admitted with ulcer is observed gradually to lose flesh and strength, without making any complaint except that he feels weak. On closer questioning, he will admit that his bowels are loose, though the dejections, in frequency, may not exceed twice a day. After a time, small clots of blood appear in the stools, which are of a light yellow or reddish colour, of thin pultaceous consistence, and very fœtid. In more acute cases, mucus and blood are discharged from the commencement of the illness, with straining and tenesmus, sense of heat, and sometimes soreness below the navel. Thirst and loss of appetite for food are attendant symptoms; the pulse is increased in frequency, and the skin is occasionally hot. At the commencement the tongue is coated; but as the disease advances, it becomes red and smooth. Emaciation progresses, the symptoms become less acute, the pain perhaps entirely ceases, but the patient is evidently losing ground; the dejections continue frequent, and œdematous swellings in the hands and feet now occur. The discharge from the bowels continues yellowish, and of a creamy consistence, or perhaps becomes almost entirely a bloody liquid. In cases attended with much anasarca, the watery accumulations are frequently removed a short period before death, by numerous liquid stools; and the bodies in these cases exhibit a degree of emaciation I have never elsewhere met with." 52.

The post mortem appearances are somewhat puzzling. In several cases of speedy demise, "the lesion discovered was slight and recent—whilst in others of great standing, it has appeared difficult to understand how life could be supported under such extensive disorganizations." Emaciation is excessive—the small intestines are found pale and thin, and seldom vascular. The colon is unaltered externally; and its mucous membrane exhibits alterations varying from a slight inflammatory blush to deep ulcerations, or even gangrene. The rectum not unfrequently displayed a greater change of structure than the colon.

"Again, without much increased vascularity of the villous surface, the whole would be studded with numerous minute ulcerations, evidently of a chronic character: in others the villous coat would have a dark uniform purplish blush; the membrane thickened, irregular, and soft, and either in the colon or rectum, generally in both, there were some deep ulcers. In one instance the ulcer was found to have penetrated the peritoneal coat of the intestine: and lastly, both omentum and colon have been found gangrenous. In a single instance the mesocolon was found vesicular; but in no instance was disease of the mesenteric glands discovered. The liver, stomach, and spleen were generally healthy. Serum to a moderate extent was usually found in the abdominal cavity; in two instances, where it existed in considerable quantity, the surface of the spleen was found white and corrugated, like skin long steeped in hot water, or pickled tripe. In the anasarcous cases, several ounces of fluid were always found in the pericardium; the viscera in the thoracic cavity were generally healthy." 54.

It is indeed astonishing to what extent ulceration of the intestines will go in chronic cases, with little apparent disturbance of the constitution. The stools will often be for days and weeks, apparently—really healthy, where numerous ulcers exist in the colon and about the ileo-cœcal valve, and where the flesh is daily diminishing.

Our author gives the result of his experience in the treatment of dysentery. He considered opium, in every shape, as injurious. In all cases where there were pain and tenderness in the abdomen, he bled, and found the blood buffed. Ipecacuan he considers the most important medicine. He gives it in doses of four or five grains, with extract of gentian. "In the more acute cases, and at the commencement of the disease, after the requisite depletion, calomel with opium, or blue pill with Dover's powder are sometimes beneficial; and mild laxatives of castor oil or rhubarb are essentially requisite in almost every case." This passage shews that the practice pursued thirty years ago is still the best in the long run.

### III. EPIDEMIC CATARRH OR INFLUENZA. By Dr. *Ward.*

It is not a little curious that the influenza which harrassed this country in the beginning of 1833, commenced, or at all events occurred, in the

Island of Java, in May, 1831, and reached Penang, in the straits of Malacca, by the middle of July, 1832. In January, 1833, it visited us, and has since gone westward, and is now probably extinguished in the back woods of America! That the disease was identical with that which occurred here, is proved by the following succinct description of it, allowing for the great difference of climate.

"The disease appeared in the form of severe catarrh, attacking suddenly, in many cases with rigor. The usual symptoms were ardent fever; great languor; sudden prostration of strength; head-ache, often violent; with heaviness over the eyebrows; severe muscular pains over the body, but more especially in the lower extremities; frequently nausea, and sometimes vomiting; harrassing and constant cough, at first unattended with expectoration; accompanied sometimes with pains in the chest; sore-throat, producing difficulty of swallowing; slight inflammation of the eyes; increased flow of tears; sneezing and copious discharge of thin acrid mucus from the nostrils." "There was considerable thirst; want of appetite in most of the cases, and the tongue was coated with a white fur. The depression of strength and spirits was considerable during the continuance of the febrile symptoms. The nights were restless, and the cough and catarrhal signs were generally most severe towards the evening, and in the mornings. These symptoms continued, with more or less severity, for two or three, seldom more than four, days, when they gradually disappeared, leaving the patient weak and languid." 125.

Thus then we see that the influenza *travelled*, as is the expression, from East to West, at a somewhat quicker rate certainly than the cholera—but still in the same course—and, we may fairly conclude, in the same manner. Was this by contagion? And if not, why not? The contagionists must answer this puzzle.

### IV. Remarks on Malignant Ulcer and Hospital Gangrene. By *J. L. Geddes*, Esq.

This we must pass over for the present, and proceed to another subject.

### V. On Abscess of the Liver in European Subjects at the Madras Presidency. By *W. Geddes*, Esq.

This is a subject of great interest in India—and even to medical practitioners in this country. The author's attention was attracted to it by the frequency of its occurrence in a body of European troops under his charge. In numerous casualties dissection showed the disease in question. "The symptoms varied greatly in almost every case previous to death; and the fatal event seemed to depend often upon the presence of peculiar affections superadded to those more immediately resulting from the collection of purulent matter." This led our author to carefully trace the history of these cases, and inquire into all the symptoms which occurred from the individual's arrival in India, up to the final period.

"On a reference to Table, No. 2, the site and size of the abscess will be observed, as found on dissection. From this table, it appears, that the most common description has been a large and solitary collection of purulent matter; and this, most generally, has been situated in the upper part of the right lobe of the liver, towards its posterior surface. Fifteen of twenty-six abscesses were placed in this situation. In two of these, the disease had penetrated the diaphragm and lungs, part of the contents of the abscess having been brought up by expectoration; and in another, the collection of matter pointed outwards between the ribs, and had been laid open some time previous to the patient's decease. In the remaining twelve, the abscess continued entire, bounded either by the substance of the liver, the ribs, or diaphragm. In three cases, the collection of matter was found near the lower margin of the right lobe, which adhered in two of them to the colon. The left lobe was, only in two instances, the seat of a solitary abscess; in one of them, the tendency of the disease was towards the concave surface, where the abscess had burst, and its matter became diffused over the abdomen: and in the other, the seat of the disease was in the upper convex portion of the lobe. In one case, there was one large abscess in the centre of the right lobe, and a small one in the left; while, in the remaining five of the 26 cases recorded, the disease consisted in a number of small abscesses, diffused through the substance of both lobes of the liver." 294.

The liver was generally protruded beyond the ribs by the distention of the abscess, giving rise to the appearance of enlargement of the organ; but there was not actual hypertrophy of the substance of the liver. The contents of the gall-bladder were, almost always, thick and tarry; or thin and glairy.

"In the large intestines, the diseased appearances were generally in proportion to the degree of dysenteric affection, immediately preceding death. In 10 cases, where there was either no irregularity, or the bowels were inclined to constipation, or the evacuations, although loose, were more of a diarrhœal nature, and generally unattended with blood, little or no disease, with the exception of some contraction of the colon, was met with. In the remaining cases, ulceration or disease was discovered in all degrees, in proportion to the previous dysentery, and varying from one or two superficial ulcers in the head of the colon, to general ulceration, with thickening of the large intestines. It may be remarked, that in all the instances, with one exception, where the intestines were found healthy, the abscess was situated in the upper and outer part of the right lobe, or deep seated in this lobe; but in the five other cases, where the collection of matter was thus situated, all had severe dysenteric symptoms in the course of their illness; and in Nos. 22 and 28, this affection was more immediately the cause of death. In one case, where the disease in the liver was in the inferior margin of the right lobe, and contiguous to the colon, there was no disease of the intestines; in another, there were only one or two superficial ulcerations, where the liver adhered; and in the third, there was more extensive ulceration. The disease of the intestines, where the left lobe was the seat of the abscess, was not very conspicuous on dissection, nor the dysenteric symptoms severe at the time of death; but in all five cases of numerous abscesses, the intestines were peculiarly affected." 297.

The symptoms of an abscess having formed in the liver were modified, partly by the site of the abscess, but chiefly from the peculiarities of constitution in the patient. No two cases presented the same phenomena, whether in their symptoms, progress, or in the final cause of death.

"There does not appear, either, any one symptom which has been constantly present in cases of this description; or which is not occasionally met with where dissection afterwards proves that an abscess in the liver had not existed. Hence, in many instances, an uncertainty in the diagnosis, the result of which is more particularly evident from the different designations given to the disease on the patient's last admission into hospital. Thus it will be seen from one of the columns of Table No. 1, that in reference to the symptoms which had chiefly attracted attention, on the patient's reporting himself, 16 only of 28 cases had been named liver or hepatic disease. Five were considered dysentery; two continued, and one intermittent, fever; two abdominal inflammation; one chronic diarrhœa, and one debility. The same circumstance would be equally evident, if the designations of the attacks preceding the fatal one were in like manner examined. From the above terms, however, as well as the table at page 291, and the fact is confirmed by an examination of the cases, it appears, that the most common and earlier symptoms of the disease are either,—firstly, some description of uneasy sensation referred to the site of the liver, or to the adjoining parts, as the thorax, abdomen, or right shoulder:—secondly, affection of the bowels, either of a dysenteric or diarrhœal nature:—and thirdly, pyrexia under different forms. Sometimes two or more of these morbid indications are combined, particularly a degree of pyrexia with either pain or dysentery: but in general, where the dysenteric symptoms are severe, there is little pain of side; and again, where the pain is urgent, there is not much annoyance from affection of the bowels.

From many of the cases, there can be little doubt entertained, that the process of suppuration in the liver may be unattended with pain. Thus in the most rapid of them, where the patient was only eight days in hospital, and it is stated in the journal, that the pain had seized him on the preceding day, and was removed, although for a time only, by venesection to the extent of two pounds of blood, and 88 leeches in the first 24 hours of his being in hospital, a large abscess, containing a pint of matter, is recorded as being found upon dissection in the right lobe of the liver. In No. 4, no pain was reported to exist in the region of the liver throughout the disease, while in Nos. 5, 8, 12, 23, and 24, none was reported until from 8 to 14 days before the death of the patient. In several other cases, the pain which has been extremely slight and transient, and in almost every instance, where the collection of matter has been found even to the extent of a quart, there have been periods of perfect immunity from this symptom, sometimes of considerable duration, while from the size of the abscess on dissection, it is impossible to conceive that the purulent matter had not existed, and been progressively increasing at these periods, or that where there had been no pain felt until shortly before the decease, the symptoms which were previously met with had not been caused by the presence of a collection of this description. These considerations may in some measure explain my having dated the commencement of the disease in some instances at so long a period prior to the death of the patient; even where there was no pain complained of in the site of the liver, and my having referred various feelings and symptoms which are found recorded, to the presence of an abscess in this organ, when dissection afterwards proved that such was ultimately the cause of death." 299.

It appears from our author, that no dependance can be placed on the site of the pain. Sometimes it was in one place, sometimes in another, and often no where. One case is so peculiar, that we shall state it.

"The patient reported himself on the third day after his illness attracted his attention, and on the 11th from his decease. He complained, on admission, of severe pain in the lower part of the right lobe of the liver, and it was felt along the edge of the ribs on pressure, but he could draw his breath pretty freely, and lie on his right side; having a sensation of a great weight however in turning over to his left. He had no pain in the shoulder. On the 5th day from admission, he was free from any uneasy sensation, excepting that of a feeling of weight at his breast, as if, according to his own account, he had eaten suet, and had a desire to vomit. On the following day, this sensation was changed to a degree of dyspnœa, imputed to weakness, a feeling of being hot and oppressed, and until two days before his death this feeling is recognized by the expression of 'a sense of oppression and clogging in the breast,' or 'at the stomach,' or

'across the chest,' or 'a feeling of a ball at the scrobiculus cordis, which on inspiration is conceived to be passing upwards.' During the last two days of his life, his sense of anxiety was attended with general pains in his limbs, and a sensation of deadness or coldness in them, which caused him to scream." 307.

The large intestines appear to peculiarly sympathize with the state of the liver. Few of the cases of hepatic abscess have been unattended by bowel-complaint.

"The constitutional disorder attendant upon these cases is undoubtedly to be considered as a pure hectic fever, which varies in little or no degree from that described by the most experienced writers in Europe, as attendant on purulent collections situated in similar circumstances with those which form the subject of this paper. In a few instances the symptoms of hectic have been extremely mild for some time, or have been unattended with frequency of pulse, or heat of skin, the only indications of general disorder being the low exhausted state of the patient, some irritability of the pulse, want of appetite or sleep, and an increasing emaciation. In other cases again, by causes of excitement, or other means, a degree of synochal fever appears to become connected with the hectic, a circumstance which is often found on the admission of the patient into hospital, requiring active measures for his relief, but, as may be inferred, having no effect in the prevention of an abscess, this being already formed. In a few instances, these affections have put on more the features of a continued fever, similar to that of irritation, the pulse continuing small and frequent for a few days; at some particular period, with little appearance of exacerbation or remission. The constitutional disorder, however, in general assumes the aspect of a paroxysmal fever. Under its form, the febrile accessions have been found to occur in some instances with a sudden attack of shivering, vomiting, and purging, and the symptoms diminished gradually, in a day or two, the patient being left, comparatively speaking, free from fever, the pulse gradually declining to the rate at which it had been previous to the febrile paroxysm. These accessions occur at intervals of various duration, and often put on the appearance of relapses of common intermittent or remittent fever. At other periods such exacerbations are less marked, the chief symptoms being rigors occurring at irregular intervals, generally with some increase of frequency of the pulse, and alternating with sweatings, which have been usually remarked at some period of the night. These sweatings have been in some individuals excessive, the perspiration being stated to be pouring off them like rain, and in one or two instances of this description, there has been considerable sinking of the vis vitæ at the same time.

In the more chronic cases, however, the most usual description of hectic consisted in a degree of fever, evidenced by an increased frequency of the pulse, at some period of the afternoon or evening, with heat of skin, and this was also observed occasionally to have terminated in perspiration. These daily paroxysms were at times broken in upon by either of the forms of hectic above-mentioned, while upon other occasions, after ceasing to be manifested for several days they were again observed. Under all these forms of hectic, the pulse is very generally recorded as weak or thready, or irritable, very seldom having a full soft feel; and having a daily or other range, according to the state of the fever. At the same time, as the disease proceeded to its fatal termination, there was a greater general frequency of the pulse, subject to be occasionally further increased on the accession of the hectic paroxysms. There was a great diversity however in the degree of frequency of the pulse in each individual; in some there being little general acceleration for a long period of the disease, while with others, it ranged constantly above a hundred for some weeks previous to the patient's death. The pulse has also been observed to be irregular, at times, in the progress of some of the cases.

Along with the attacks of hectic, the countenace of the individual generally loses its florid aspect, becoming either of a pallid, leucophlegmatic or sallow hue. In one case, it has been mentioned, that the patient became somewhat jaundiced with almost every accession of fever; and, in No. 8, there was an affection of a like nature from the fourth day preceding the disease.

Debility and emaciation succeed according to the length to which the disease is extended, and in a few instances, where this has been of some duration, these symptoms have eventually been very conspicuous, and œdema of the legs and right arm has been met with." 329

One more short extract, and we shall close this article.

"Inflammation of the liver has long been distinguished into the acute and chronic hepatitis; and there is reason for a distinction in the inflammatory diseases of this organ, although, not from being, I am inclined to think, more or less attended with inflammatory symptoms as has been generally understood by these terms of chronic and acute, but inasmuch as one is an acute inflammation, occurring most probably in the peritoneal covering of the liver, and having a tendency to terminate in resolution or adhesion, while the other is only an occasional aggravation of a disease which has existed for some time, most probably in the parenchyma of this viscus, and which may continue its progress after the more inflammatory symptoms have been subdued. The latter, in short, is the disease under which inflammation of the liver most usually proves fatal, and is that which, under its different aspects appears to have affected the greater number of the cases above described.

In the treatment of each of these varieties of disease of the liver, it would appear useful to recollect the above distinction, even where the inflammatory symptoms of each seemed of equal severity. In the first, antiphlogistic measures may be used to their fullest extent with benefit, and with these the affection of the mouth by mercury to ptyalism will remove the disease. In the latter indicated either by previous bad health, the constitution of the patient, or symptoms such as have been described to have attended the above cases, it would seem advisable to bear in mind the necessity of reducing the strength as little as possible; and the propriety of exhibition of mercury, which in few cases excites a free salivation, may be questionable. The analogous case of phthisis pulmonalis ought, I think, to be held in view, particularly in the more chronic cases of this hepatic disorder; and the indications for the treatment generally should be, in the 1st place, attention to urgent symptoms; and 2dly, to change the tendency to the formation of purulent matter, and to induce an absorption of that already formed, by diminishing the hectic fever, and strengthening the patient's constitution. This, I imagine, is not to be done by means of medicine, although the quinine has been found serviceable in both these respects; but chiefly by the gradual aid of change of air and scene, gentle exercise, and a sea voyage, with peculiar attention to the use of a mild, nourishing, slightly stimulating diet, such as is recommended to consumptive individuals, or those suffering from hectic fever, produced by causes similar to an abscess of the liver." 336.

We must now conclude our first notice of this volume. In our next Number we shall proceed with our analysis, and hope to do justice to the professional exertions of our Oriental brethren.

